# Safety of vacuum-assisted biopsy/mammatome guided, non-operative management of B3 lesions without atypia: a 7-year follow-up study

**DOI:** 10.1186/bcr3792

**Published:** 2015-11-05

**Authors:** Alex Wilkins, Peter Kneeshaw, Penelope McManus, Kartikae Grover, Caroline Bradley, Ayesha Rahman, Anne Hubbard

**Affiliations:** 1 Castle Hill Hospital, Cottingham, UK; 2 Hull York Medical School, Hull, UK

## Introduction

B3 management balances safe treatment of potential malignancy against the morbidity of surgical excision of benign lesions. Vacuum-assisted biopsy (VAB) increases diagnostic accuracy, removing some lesions entirely without surgery. Few follow-up data are available to assess the safety and effectiveness of this approach.

## Methods

A total of 215 patients with B3 biopsies without atypia were identified using Labcentre histopathology codes at a single centre. Hospital and NBSS records were analysed to identify patients who were treated with VAB and mammographic surveillance alone and to determine outcome over a follow-up period of 52−149 months (median 85). Local Labcentre and regional Pathlinks histopathology records were independently checked. Mammograms of ipsilateral re-presentations were reviewed by a consultant radiographer and consultant radiologist to determine whether lesions developed at the site of B3 biopsy.

## Results

Twenty per cent had excision biopsy (42/215) of which <5 % (2/42) contained carcinoma. A total of 144 patients had VAB which identified 30 high-risk cases analysed separately (DCIS, B4 or atypia). In total, 114 B3 lesions without atypia (on either core biopsy or VAB) were followed mammographically after VAB with no surgical intervention. Four patients re-presented to the service with malignancy; 37, 38, 41 and 67 months after VAB. Sixty-one per cent (69/114) of individuals were screened locally 2012−2015.

## Conclusion

VAB of B3 biopsies without atypia appears to be safe with no representations in the first 3 years and overall carcinoma and DCIS incidence of 3.5 % over 7 years (4/114). National guidance on B3 lesion management is required.

